# The Care for Non-COVID-19 Patients: A Matter of Choice or Moral Obligation?

**DOI:** 10.3389/fmed.2020.564038

**Published:** 2020-10-29

**Authors:** Bashar Hassan, Thalia Arawi

**Affiliations:** ^1^Faculty of Medicine, American University of Beirut, Beirut, Lebanon; ^2^American University of Beirut Medical Center, Beirut, Lebanon

**Keywords:** COVID-19, coronavirus, SARS-CoV-2, non-COVID-19 patients, ethics, elective surgeries, psychological interventions, mental health

## Introduction

A male patient Sam (not his real name), suddenly unable to walk, presented to Addington Hospital in Durban, South Africa, a couple of days before the president declared a national state of disaster due to SARS-CoV-2. An MRI scan at Inkosi Albert Luthuli Hospital was needed to establish the suspected diagnosis of Tuberculosis (TB); however, he was told he had to wait for months or even a year, due to the huge strain the aforenamed hospital was facing because of the corona virus. Therefore, Sam was not provided with a clear diagnosis, and thus no proper treatment. He was told that he will not be able to walk again and sent back home to an overcrowded hostel where his family and hundreds of other underprivileged people could contract the disease if it really was TB (1). From South Africa to Canada, due to the massive burden of COVID-19 on the health care systems, Sydney Loney had her mastectomy, which was originally scheduled in mid-March, postponed indefinitely (2). Due to the scarce medical resources SARS-CoV-2 has left hospitals with, patients with deadly infectious diseases like TB, cancers that can metastasize, and numerous other conditions, are being denied medical care. In addition, thousands of elective surgeries are being canceled (3). In this article, we shed light on the ethical challenges imposed by SARS-CoV-2 regarding non-COVID-19 patients and raise the possibility of establishing more considerate regulations and specific psychological interventions for this subset of patients.

SARS-CoV-2 is the newly discovered infectious virus responsible for Coronavirus disease (COVID-19) (4). Owing to the immense burden of COVID-19 pandemic on health care systems, the Centers for Medicare and Medicaid Services, the U.S. Surgeon General, the American College of Surgeons (ACS) and many other medical specialties and societies have put guidelines for the temporary cancelation of elective surgeries (5, 6). The ACS has even thanked the surgeons who already stopped performing them (6). These recommendations and guidelines are still being constantly updated as to how surgeons should choose whether or not to perform a certain operation (6). Over decades, the scientific literature has been flooding with incessantly updated guidelines on providing the best medical care for patients, as well as conducting surgeries. It is morally perplexing, as a budding medical professional and junior physician, to see guidelines released to restrict medical care and delay surgeries instead (5, 6).

## Ethical Challenges Related to Non-COVID-19 Patients

COVID-19 and the aforementioned guidelines emanating from it challenge the basic ethical principles of medicine such as the universal right to healthcare, beneficence, non-maleficence, justice, to mention but a few core ones (5, 6). On the other hand, having specific guidelines for elective surgeries (5, 6), may ensure equality among non-COVID-19 patients, only if all hospitals agree to abide by these guidelines (which is very unlikely). Many hospitals do not follow the aforementioned published guidelines, which raises the possibility of unfair treatment and different handling of two patients with the same medical condition requiring the same “elective” surgery, if they had gone into two different hospitals, placing the patient who have been denied surgery at a disadvantage. It also puts the healthcare institution, which does not offer a valid moral justification for its “triaging,” in a difficult situation in terms of trust and reputation.

Aside from published guidelines, many other factors have contributed to the cancellation of elective surgeries, and some perhaps were contributing factors to establishing these guidelines. For example, the shortage of PPEs and beds necessary for operations, and even medical personnel who have been summoned to work on COVID-19 floors, has put COVID-19 patients at risk of not receiving the appropriate medical care (7). Also, some insurance companies are not paying except for urgent operations, which may in turn delay elective surgeries of non-COVID-19 patients. Some of these patients may themselves refrain from seeking medical care fearing that they may contract the COVID-19 virus.

Allocating hospital resources to COVID-19 patients should not mean leaving potentially fatal or impedingly dangerous cases untreated. This may perhaps be achieved through more considerate regulations that give equal, or at least greater, considerations for non-COVID-19 patients. We see this particularly important building on the fact that delaying medical treatment for non-COVID-19 patients may be more harmful to health care systems on the long run with the rising numbers of non-COVID-19 patients, thus placing health care systems in an endless cycle of medical care shortage. To explain this further, we take TB as an example. TB is the number 1 cause of death from an infectious disease (8); therefore, both diseases, COVID-19 and TB, can be fatal and are contagious. Untreated TB patients might further spread the disease, ending up with more non-COVID-19 patients in need of medical care, and thus exacerbating the existing situation. Hence, regarding TB as less important than COVID-19, in our opinion is counterintuitive, as it defeats the purpose of reserving medical care for COVID-19 patients and may in fact increase the burden on health care systems and the risk of an impending health catastrophe.

This is particularly important in certain populations like that of Nigeria. Experts have declared that the focus of Nigeria should be to tackle TB rather than COVID-19, as the former is worse than the latter (9). They based their recommendations on the fact that TB kills over 3,000 people daily in Nigeria, at a time when COVID-19 kills 60 people a day (9). The Head of TB unit at the World Health Organization (WHO) expressed that the treatment of TB in Nigeria is hugely lacking proper funding, which mainly comes from US agencies (9). Because many funds are diverging into COVID-19 research and resources, this is another reason COVID-19 exacerbates other conditions, like TB.

Following the same line of thought, the COVID-19 pandemic has strongly contributed to delaying acute care of strokes and myocardial infarctions, routine monitoring, preventive protocols, childhood vaccinations, and cancer, diabetes, and lipid disorders treatments (10). Such delays may have significant future population-based consequences (10), what we note will lead to a health catastrophe. Although the above may be seen as a public health priority, to us focusing on a blooming disease rather than a greater burden for a certain population, or ending up with people dying because of delaying proper screening or preventive measures, is ethically challenging as it undermines the basic bioethics principles of medicine and the notion of humane medicine.

The consequences of curtailing surgeries and medical care are not only limited to the present health risks we are currently facing, but also to the future health risks that will arise from canceling or refraining from a plethora of surgical procedures, screenings, and prevention. These anticipated health risks will most probably not only be physical, but also mental.

## Mental Health of Non-COVID-19 Patients

The specter of COVID-19 has been hovering for months now. Many family members are not being able to see their hospitalized loved ones. While some countries offer virtual solutions, many find this a cold way of “being” with their loved ones, leading to feelings of helplessness. Other persons are subjected to great distress for not holding proper funerals and burial rituals for their beloved family members (11). Healthcare workers are under a lot of mental and physical stress; some even opted to end their lives (12). Adding those to the social distancing afflicted by the Coronavirus, we see the world going into a pandemic of a mental health nature.

In light of an already deteriorating mental health, this impact of COVID-19 is often exacerbated in those who are sick yet denied medical care, proper treatment, and elective surgeries. Psychological interventions have been designed to deal with COVID-19 patients, survivors, and other people in quarantine (13). To our knowledge, however, there is scarce data on the psychological interventions for non-COVID-19 patients who have been denied elective surgeries, proper medical care, access to the hospital, etc. We would like to encourage greater consideration of this particular subtype of patients in newer guidelines and raise the possibility of designing specific psychological interventions for them to protect their threatened mental health and psychological well-being. We contend that this is an issue of paramount importance that should be addressed by the national bioethics committees of different countries, as mental health is as important as physical health.

As a conclusion, to mitigate consequences of the COVID-19 pandemic, regular monitoring, screening tests, preventive measures, and quality care should resume as soon as possible to non-COVID-19 patients who have been denied proper care during the pandemic. This may be a challenging task as it requires that hospitals keep a track record and contact the “rejected” or “postponed patients” who might have undergone the treatment/procedure elsewhere. Also, psychological interventions should target COVID-19 patients, survivors, those who lost their loved ones, and non-COVID-19 patients whose management has been delayed. We reckon that all patients have the same right to proper medical evaluation, care, and treatment. Although the new guidelines prioritize COVID-19 patients, other patients are still patients, and we as health care professionals have a duty toward all.

## Author Contributions

BH is the main contributor and writer and edited accordingly. TA revised the article, raised several remarks, titled the article, and made the final edits. All authors contributed to the article and approved the submitted version.

## Conflict of Interest

The authors declare that the research was conducted in the absence of any commercial or financial relationships that could be construed as a potential conflict of interest.
